# Amphetamine Containing Dietary Supplements and Acute Myocardial Infarction

**DOI:** 10.1155/2016/6404856

**Published:** 2016-07-19

**Authors:** Julio Perez-Downes, Abdulwahab Hritani, Candice Baldeo, Patrick Antoun

**Affiliations:** ^1^Department of Internal Medicine, University of Florida College of Medicine-Jacksonville, Jacksonville, FL 32209, USA; ^2^Department of Cardiovascular Medicine, University of Florida College of Medicine-Jacksonville, Jacksonville, FL 32209, USA

## Abstract

Weight loss is one of the most researched and marketed topics in American society. Dietary regimens, medications that claim to boost the metabolism, and the constant pressure to fit into society all play a role in our patient's choices regarding new dietary products. One of the products that are well known to suppress appetite and cause weight loss is amphetamines. While these medications suppress appetite, most people are not aware of the detrimental side effects of amphetamines, including hypertension, tachycardia, arrhythmias, and in certain instances acute myocardial infarction. Here we present the uncommon entity of an acute myocardial infarction due to chronic use of an amphetamine containing dietary supplement in conjunction with an exercise regimen. Our case brings to light further awareness regarding use of amphetamines. Clinicians should have a high index of suspicion of use of these substances when young patients with no risk factors for coronary artery disease present with acute arrhythmias, heart failure, and myocardial infarctions.

## 1. Introduction

Amphetamines are widely known to cause appetite suppression and encourage weight loss. Their other known side effects are hypertension, tachycardia, arrhythmias, and myocardial infarctions [1]. Herein we describe the case of a 35-year-old female who was undergoing a weight loss regimen of daily exercise with a dietary supplement who subsequently suffered an acute myocardial infarction and was found to test positive for amphetamines.

## 2. Case Presentation

**Figure 1:**
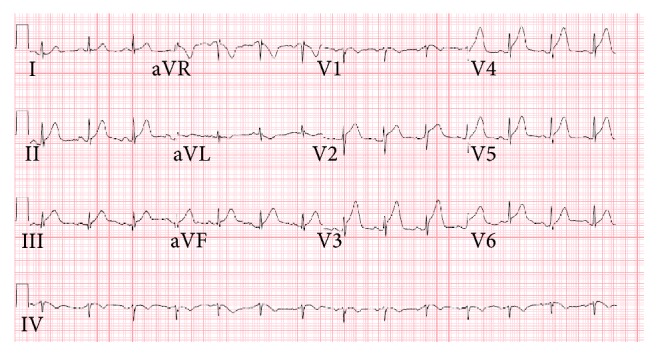
Presenting EKG showing inferior-lateral ST segment elevation.

**Figure 2:**
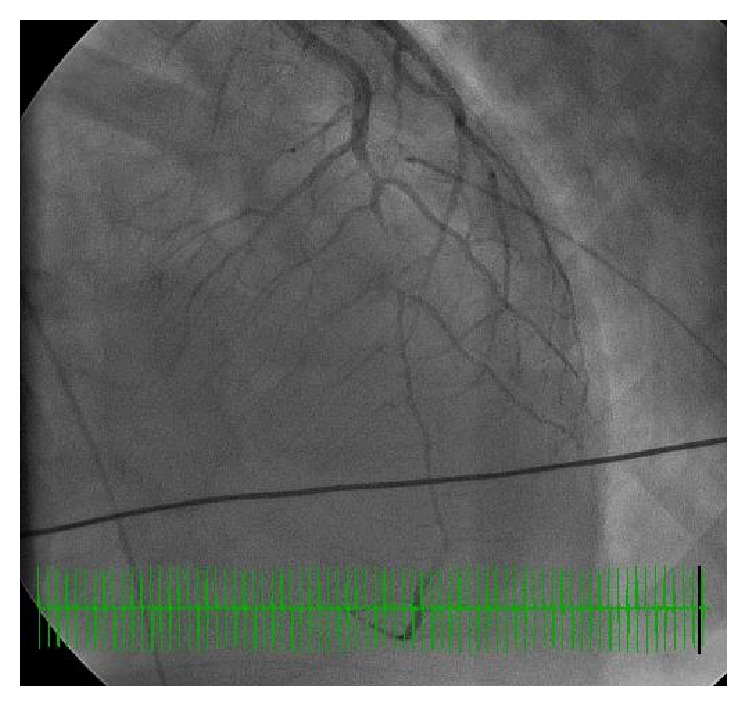
Left heart catheterization representing 99% occlusion of mid-distal LAD.

**Figure 3:**
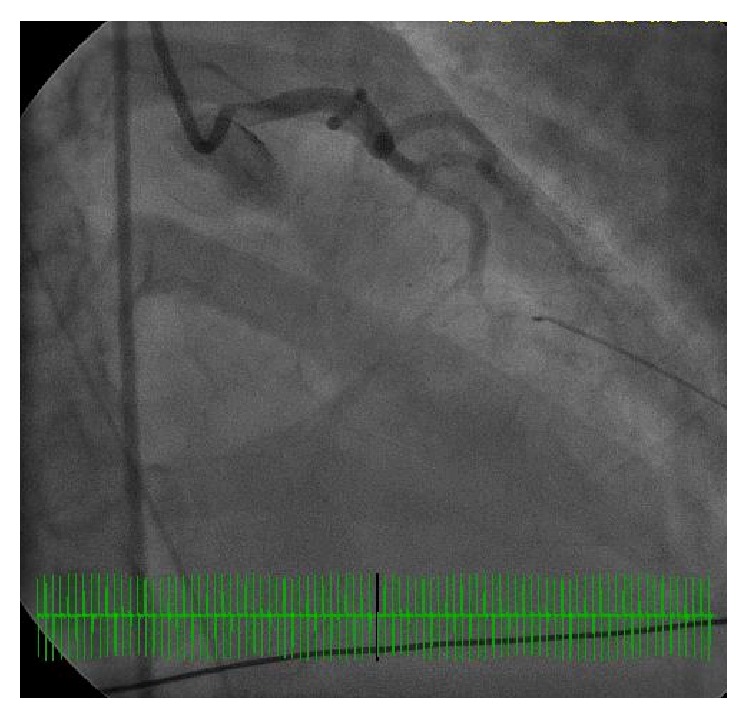
Left heart catheterization representing 99% occlusion of mid-distal LAD.

**Figure 4:**
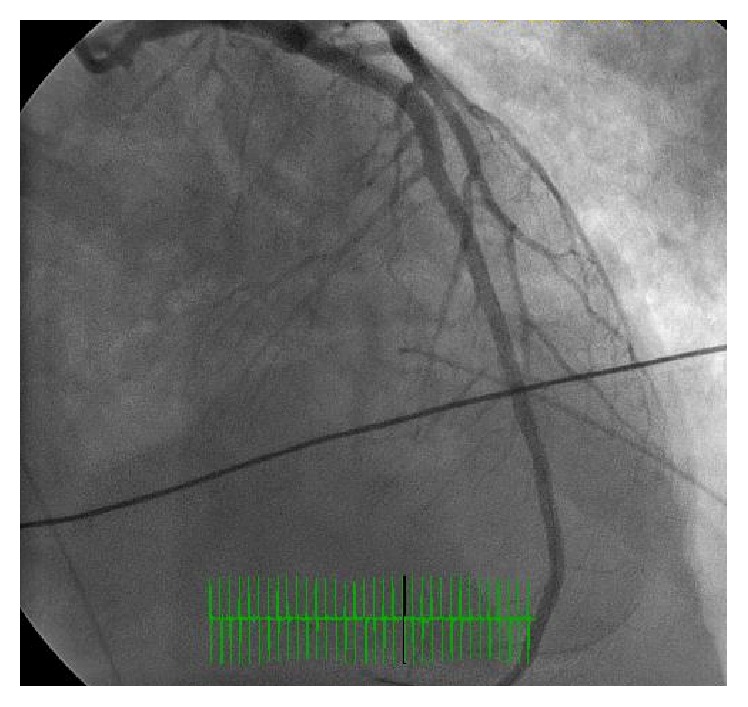
Left heart catheterization image postimplantation of two overlapping drug eluting stents, depicting TIMI 3 flow.

A 35-year-old African American female with no prior history of coronary artery disease and no significant family history presented with sudden onset of exertional chest discomfort with radiation to the back. The patient became unresponsive shortly after arrival to the emergency department and was subsequently found to be in ventricular fibrillation-cardiac arrest (V-fib). The patient was in V-fib for 6 minutes, with conversion after electrical cardioversion and subsequent development of PEA-arrest for a total of 4 minutes. Repeat EKG after return of spontaneous circulation demonstrated inferolateral STEMI (Figure 1). The patient received tenecteplase and heparin prior to urgent transfer to the catheterization laboratory. Left heart catheterization showed 99% thrombotic occlusion of mid-distal LAD (Figures 2 and 3). Two overlapping drug eluting stents (Xience RX 2.5 mm and Xience RX 2.75 mm) were placed in the distal LAD, and TIMI III flow was achieved (Figure 4). Due to severely decreased ventricular function with ejection fraction of 10–15%, the patient received ventricular support with an Impella, with discontinuation soon after secondary to hemolysis. Further evaluation revealed positive toxicology screen for amphetamines. Pertinent laboratory results for possible autoimmune causes of the acute myocardial infarction were negative, including antinuclear antibody (ANA), ribonucleoprotein antibody (RNP), RA latex turbid, anti-chromatin IgG antibodies, SSA (Ro) and SSB (La) antibodies, and double stranded DNA antibody (dsDNA).

The patient's cardiac function recovered with medical management. Subsequent transthoracic echocardiograms revealed improved ejection fraction to 60–65% after 11 days and a new finding of left ventricular apical thrombus. The patient received anticoagulation with intravenous heparin, as well as continuous treatment with dual antiplatelet therapy with aspirin and clopidogrel.

The patient remained in the coronary care unit (CCU) for a total of 17 days. CCU course was further complicated by development of pulmonary edema with diffuse alveolar hemorrhage and developing MRSA and pseudomonas pneumonia.

Due to the need for prolonged mechanical ventilation, the patient received a tracheostomy and continued to improve in terms of her pulmonary function while treated with antibiotics for ventilator-associated pneumonia. Her neurological status improved significantly, and on interview, she denied any use of Adderall, amphetamines, or illicit drugs that could have precipitated this event. She reported that recently she had increased her level of physical activity in order to lose weight and was supplementing such efforts with the addition of a natural weight loss dietary supplement.

## 3. Discussion

Amphetamine use is strongly associated with coronary artery disease [2]. The immediate cardiovascular effects of amphetamine use include tachycardia and hypertension, both of which are caused by the increase in circulation of catecholamines. These can lead to life-threatening arrhythmias and enhancement of coronary vascular tone, increase platelet aggregation, and ultimately promote plaque rupture with subsequent development of an acute myocardial infarction [1, 3]. The mechanism of myocardial injury due to amphetamine use is believed to be acute coronary vasospasm, with subsequent decreased perfusion and development of an acute myocardial infarction. Chronic use of amphetamines can also lead to accelerated atherosclerosis and increased thrombogenicity [4], both of which can lead to thromboocclusive acute coronary syndromes in young individuals. Bashour reported the first documented case of intracoronary thrombus as the culprit of acute myocardial infarction in a patient with amphetamine abuse. They postulated this increase in thrombogenicity to be secondary to catecholamine-induced platelet aggregation [5]. Westover and colleagues, in a cross-sectional study which evaluated the link between amphetamine abuse and incidence of acute myocardial infarction, revealed a significant association with amphetamine use and acute myocardial infarction in young adults (adjusted odds ratio = 1.61; 95% CI = 1.24–2.04, *p* = 0.0004) [6].

There are multiple cases reported in the literature involving the development of an acute myocardial infarction due to amphetamine abuse. Chang and colleagues reported an unusual case of a silent ST elevation myocardial infarction following amphetamine use in a 61-year-old diabetic patient. In their case, the patient presented to the hospital without chest pain and normal cardiac enzymes; however, EKG revealed ST elevations in the inferior leads with reciprocal changes in the precordial leads. Subsequent percutaneous coronary angiography revealed total occlusion of the posterior-lateral segment of the right coronary artery. On further history, the patient had reported abusing amphetamines via inhalation prior to presentation [7]. In a similar case, Waksman and colleagues reported the incidence of an acute anterior wall myocardial infarction in a 31-year-old patient who was using amphetamine intravenously and presented to the hospital with generalized discomfort after 4 doses 48 hours prior to presentation [8]. On this particular case, myocardial infarction was diagnosed via electrocardiogram changes which were reported as T wave inversions in inferior and anterior leads, with subsequent transition to a new left bundle branch block in a repeat electrocardiogram 5 minutes after the prior. Conservative treatment was instituted and the patient subsequently transferred to the intensive care unit. Transthoracic echocardiogram 3 days after admission revealed decreased anterior wall motion as well as a reduced ejection fraction at 25%. The patient was unable to undergo cardiac catheterization due to leaving the hospital prematurely [8]. Watts and McCollester reported the case of a 23-year-old patient who presented to the emergency room with abdominal pain and generalized malaise less than 24 hours after inhalation of amphetamines. Electrocardiogram revealed ST elevations in the precordial leads V1–V4, a junctional rhythm, and a complete heart block, with troponin I noted acutely elevated. The patient underwent cardiac catheterization which revealed normal coronaries and a decreased ejection fraction of 15–20% [9]. Subsequent clinical course included evaluation with a transesophageal echocardiogram revealing ventricular asynergy, placement of a dual chamber pacemaker, and a follow-up transthoracic echocardiogram revealing an improved ejection fraction of 35–40% and electrocardiogram revealing resolution of ST elevation [9]. An additional case reported in the literature by Furst and colleagues involves the case of a 41-year-old patient who presented to the hospital after use of intranasal methamphetamine with chest pain [3]. Electrocardiogram revealed ST elevations in II, III, and AVF, with reciprocal changes in the precordial leads. The patient was initially treated with thrombolytics, with resolution of ST elevations and chest pain, but suffered a recurrence of chest pain 24 hours after treatment. Cardiac catheterization was performed which showed subtotal occlusion of the mid-segment of the right coronary artery, with the patient undergoing percutaneous coronary intervention [3]. This case also illustrates how despite treatment the risk of subsequent myocardial infarction after amphetamine use remains a real concern. Turnipseed and colleagues, in a study that aimed at determining the frequency of acute coronary syndrome in patients presenting to the hospital with chest pain after methamphetamine use, concluded that acute coronary syndrome is common in patients presenting with chest pain after methamphetamine use as well as the fact that a normal electrocardiogram does not necessarily rule out the possibility of myocardial infarction in patients known to be methamphetamine abusers [10].

One of the side effects of amphetamine use is decreased appetite, a side effect that is desirable to some patients. The realization of such effect and the potential for inducing weight loss led to the introduction of amphetamines as appetite suppressants in the 1950s and development of the combination of Phentermine and Fenfluramine in the 1990s [11]. The medication was approved and widely used in the early 1990s due to its significant effect in weight reduction. However, due to subsequent reports of increased cardiovascular events, the drug was withdrawn from the market in 1997 [12]. With the increased use of amphetamines for treatment of Attention Deficit Hyperactivity Disorder and Adult Attention Deficit Disorder, there has been a substantial increase in their abuse due to the desirable side effect of decreased appetite and weight loss. In view of this side effect profile, manufacturers of dietary supplements marketed to promote weight loss are including *β*-Methylphenethylamine (B-Methyl), a positional isomer of amphetamine with a similar profile effect as amphetamines [13], in their dietary supplements. This compound, which is similar in structure and composition to amphetamine, can be detected in routine toxicology screens as amphetamine.

In view of the positive toxicology screen for amphetamines and the lack of history of abuse or use by our patient, we propose the notion that weight loss dietary supplements in fact may contain amphetamines or amphetamine-like substances. With the popularity of such products among patients searching for aids in weight loss, there is a possibility that a portion of the population who regularly use these products might be exposed to unregulated levels of amphetamines or amphetamine-like substances. The acute and chronic consequences of use of these substances can be detrimental to patients, as they are at a higher risk for acute myocardial infarctions, increased risk of accelerated atherosclerosis, and early development of cardiac dysfunction due to recurrent myocardial injury [1, 4, 5, 12].

## 4. Conclusion

Our case illustrates how inadvertent use of amphetamines by patients with no history, risk factors, or significant family history of coronary artery disease can be the culprit for life threatening events. Patients often struggle with weight management, looking for alternatives to supplement their efforts to lose weight. Without proper disclosure and recent trend of the addition of amphetamines to dietary supplements [13], it is important to educate patients and maintain a high index of suspicion when young adults present with acute myocardial infarction and have no history or laboratory values significant for illicit drug use. The detrimental effects of the use of such substances can be seen in the acute phase by myocardial infarctions and left ventricular dysfunction [3, 9] and potentially in the chronic phase by subsequent valvular heart disease and congestive heart failure [12].
